# Cognitive behavioural therapy for insomnia in patients with rheumatoid arthritis: protocol for the randomised, single-blinded, parallel-group Sleep-RA trial

**DOI:** 10.1186/s13063-020-04282-6

**Published:** 2020-05-29

**Authors:** K. M. Latocha, K. B. Løppenthin, M. Østergaard, P. J. Jennum, R. Christensen, M. Hetland, H. Røgind, T. Lundbak, J. Midtgaard, B. A. Esbensen

**Affiliations:** 1 Copenhagen Center for Arthritis Research (COPECARE), Center for Rheumatology and Spine Diseases, Rigshospitalet, Glostrup, Denmark; 2 Department of Oncology, Research unit for Cancer Late Effect, CASTLE, Rigshospitalet, Copenhagen, Denmark; 3 Department of Clinical Medicine, Faculty of Health and Medical Sciences, University of Copenhagen, Copenhagen, Denmark; 4 Danish Center for Sleep Medicine, Department of Clinical Neurophysiology, Rigshospitalet, Glostrup, Denmark; 5 Musculoskeletal Statistics Unit, The Parker Institute, Bispebjerg and Frederiksberg Hospital, Frederiksberg, Denmark; 6 Research Unit of Rheumatology, Department of Clinical Research, Faculty of Health Sciences, University of Southern Denmark, Odense, Denmark; 7 Center for Rheumatology and Spine Diseases, Rigshospitalet, Glostrup, Denmark; 8 The University Hospitals Centre for Health Research, Rigshospitalet, Copenhagen, Denmark; 9 Department of Public Health, Faculty of Health and Medical Sciences, University of Copenhagen, Copenhagen, Denmark

**Keywords:** Inflammatory arthritis, Insomnia, Sleep disturbance, Cognitive behavioural therapy for insomnia, Non-pharmacological treatment, Polysomnography, Actigraphy, Fatigue, Impact of rheumatoid arthritis, Health-related quality of life

## Abstract

**Background:**

More than half of patients with rheumatoid arthritis complain of insomnia, which is predominantly treated with hypnotic drugs. However, cognitive behavioural therapy for insomnia is recommended as the first-line treatment in international guidelines on sleep. Patients with rheumatoid arthritis suffer from debilitating symptoms, such as fatigue and pain, which can also be linked to sleep disturbance. It remains to be determined whether cognitive behavioural therapy for insomnia can be effective in patients with rheumatoid arthritis. The aim of the Sleep-RA trial is to investigate the efficacy of cognitive behavioural therapy for insomnia on sleep and disease-related symptoms in patients with rheumatoid arthritis. The primary objective is to compare the effect of cognitive behavioural therapy for insomnia relative to usual care on changes in sleep efficiency from baseline to week 7 in patients with rheumatoid arthritis. The key secondary objectives are to compare the effect of cognitive behavioural therapy for insomnia relative to usual care on changes in sleep onset latency, wake after sleep onset, total sleep time, insomnia, sleep quality, fatigue, impact of rheumatoid arthritis and depressive symptoms from baseline to week 26 in patients with rheumatoid arthritis.

**Methods:**

The Sleep-RA trial is a randomised controlled trial with a two-group parallel design. Sixty patients with rheumatoid arthritis, insomnia and low-to-moderate disease activity will be allocated 1:1 to treatment with cognitive behavioural therapy for insomnia or usual care. Patients in the intervention group will receive nurse-led, group-based cognitive behavioural therapy for insomnia once a week for 6 weeks. Outcome assessments will be carried out at baseline, after treatment (week 7) and at follow-up (week 26).

**Discussion:**

Data on treatment of insomnia in patients with rheumatoid arthritis are sparse. The Sleep-RA trial is the first randomised controlled trial to investigate the efficacy of cognitive behavioural therapy for insomnia in patients with rheumatoid arthritis. Because symptoms of rheumatoid arthritis and insomnia have many similarities, we also find it relevant to investigate the secondary effects of cognitive behavioural therapy for insomnia on fatigue, impact of rheumatoid arthritis, depressive symptoms, pain, functional status, health-related quality of life and disease activity.

If we find cognitive behavioural therapy for insomnia to be effective in patients with rheumatoid arthritis this will add weight to the argument that evidence-based non-pharmacological treatment for insomnia in rheumatological outpatient clinics is eligible in accordance with the existing international guidelines on sleep.

**Trial registration:**

ClinicalTrials.gov: NCT03766100. Registered on 30 November 2018.

## Administrative information

The numbers in curly brackets in this protocol refer to the Standard Protocol Items: Recommendations for Interventional Trials (SPIRIT) checklist item numbers. The order of the items has been modified to group similar items (see http://www.equator-network.org/reporting-guidelines/spirit-2013-statement-defining-standard-protocol-items-for-clinical-trials/).

**Table:** 

Title {1}	Cognitive behavioural therapy for insomnia in patients with rheumatoid arthritis: protocol for the randomised, single-blinded, parallel-group Sleep-RA trial
Trial registration {2a and 2b}	ClinicalTrials.gov: NCT03766100. Registered on 30 November 2018.
Protocol version {3}	15 January 2020. Version 1.
Funding {4}	The Sleep-RA trial is funded by grants from the Danish Rheumatism Association, Rigshospitalet, Lundbeckfonden, the Novo Nordisk Foundation, Danish Nurses Organization and Toyota-Fonden, Denmark. The Parker Institute, Bispebjerg and Frederiksberg Hospital (RC) is supported by a core grant from the Oak Foundation (OCAY-18-774-OFIL).
Author details {5a}	^1^ Copenhagen Center for Arthritis Research, Center for Rheumatology and Spine Diseases, Rigshospitalet, Glostrup , Denmark. ^2^ Research unit for Cancer Late Effect, Department of Oncology, Rigshospitalet, Copenhagen, Denmark. ^3^ Department of Clinical Medicine, Faculty of Health and Medical Sciences, University of Copenhagen, Copenhagen, Denmark. ^4^ Danish Center for Sleep Medicine, Department of Clinical Neurophysiology, Rigshospitalet, Glostrup, Denmark. ^5^ Musculoskeletal Statistics Unit, The Parker Institute, Bispebjerg and Frederiksberg Hospital, Frederiksberg, Denmark. ^6^ Research Unit of Rheumatology, Department of Clinical Research, Faculty of Health Sciences, University of Southern Denmark, Odense, Denmark. ^7^ Center for Rheumatology and Spine Diseases, Rigshospitalet, Glostrup, Denmark. ^8^ The University Hospitals Centre for Health Research, Rigshospitalet, Copenhagen, Denmark. ^9^ Department of Public Health, Faculty of Health and Medical Sciences, University of Copenhagen, Copenhagen, Denmark.
Name and contact information for the trial sponsor {5b}	This is an investigator-initiated trial that proceeds from: Copenhagen Center for Arthritis Research Center for Rheumatology and Spine Diseases Rigshospitalet Bente Appel Esbensen Valdemar Hansens Vej 17 2600 Glostrup Denmark + 453,863,863 Bente.appel.esbensen@regionh.dk
Role of sponsor {5c}	This is an investigator-initiated trial solely funded by non-commercial sources. Funding sources have had no role in the design of this study and will not have any role during its execution, analyses, interpretation of the data, or decision to submit results.

## Introduction

### Background and rationale {6a}

#### Rheumatoid arthritis

Rheumatoid arthritis (RA) is a chronic autoimmune inflammatory disease with a prevalence worldwide of 0.4–1.3%. Women are affected three times more frequently than men, and onset is most often at 50–60 years of age [1, 2]. Symptoms include joint swelling, pain and stiffness, and often significant disability. Disease activity is most commonly monitored with Disease Activity Score-28 C-reactive protein (DAS28-CRP) based on the number of swollen joints, the number of tender joints, serum CRP and patient global assessment [3, 4]. Improvements in early diagnosis and medical treatment with anti-rheumatic drugs have led to a reduced incidence of swollen and tender joints, pain and joint destruction [5]. Nevertheless, patients still report a wide range of symptoms with a high prevalence; among the most frequently reported are sleep disturbances, fatigue, depressive symptoms, pain, reduced functional status and health-related quality of life (HRQoL) [6–8]. In evaluating their medical treatment with antirheumatic drugs, patients indicate that sleep is one of the most important parameters. This highlights the major impact that sleep has on physical and mental well-being [9, 10].

Sleep disturbances are common in patients with RA, with a prevalence of 60–80% compared to 10–30% in the general population [11–13]. Furthermore, sleep quality can be associated with disease activity; for example, patients with moderate and high disease activity reported fewer hours asleep than patients with low disease activity or those in remission [14–17]. Sleep disturbances in patients with RA are characterised by significantly worse sleep efficiency (SE), sleep quality, sleep latency, number of awakenings, and time awake after sleep onset than healthy control groups [14, 18–20].

#### Insomnia

According to the International Classification of Sleep Disorders third edition (ICSD-3) and the Diagnostic and Statistical Manual of Mental Disorders the diagnostic criteria for insomnia are: sleep initiation or maintenance problems; inadequate opportunity and circumstances to sleep; and daytime consequences [21, 22]. The ICSD-3 duration criterion for chronic insomnia disorder is 3 months and a frequency criterion of at least three times per week [21]. Like RA, insomnia is associated with fatigue, depressive symptoms, pain, functional status and cardiovascular diseases [23–25].

The association between disease activity and sleep is acknowledged in patients with inflammatory arthritis, but the exact underlying mechanisms of insomnia in general have not been identified. Insomnia is often considered to be linked to hyperarousal, which decreases the likelihood of sleep; however, the heterogeneity in cause, symptoms, course, comorbidities and consequences complicates the establishment of a universal aetiological or pathophysiological model of insomnia [26, 27]. According to existing evidence on the pathophysiology of insomnia, insomnia is most likely to develop in people who have increased genetic risk and who experience abnormalities in neurobiological processes—vulnerabilities that may lead to hyperarousal and to psychological and behavioural processes that increase the risk for developing insomnia [27–29].

According to the conceptual “3P model” of insomnia by Spielman et al., three elements contribute to the development and maintenance of chronic insomnia: predisposing factors, precipitating events, and perpetuating mechanisms [30]. The predisposing factors include the fact that some individuals may have an increased risk of insomnia by having certain genetic predisposing factors of personal vulnerability that might contribute to a highly sensitive or malfunctioning biological sleep system or a hyperactive arousal system that interferes with sleep [31]. The time of diagnosis can be a precipitating event as being diagnosed with a chronic disease like RA can cause a cognitive and emotional reaction. These reactions can manifest as worry or depressed mood, which may also exploit the vulnerability of a highly reactive sleep system [32]. For patients already diagnosed with RA, several conditions related to their arthritis can represent precipitating events that contribute to the development of insomnia—for example, fatigue, pain, reduced functional status, flares and disease activity [33–35].

When insomnia becomes chronic, sleep is characterised by inappropriate habits and dysfunctional thoughts related to sleep, i.e. the perpetuating mechanisms. Insomnia is often linked to maladaptive strategies, such as avoidance behaviour during waking hours. Avoidance behaviour may include cancelling planned activities either because of feeling too tired or due to fear that the activities will interfere with sleep. Also, increased sleep effort is common, e.g. spending excessive time in bed and developing rigid sleep-related rituals. In response to insomnia, some people also develop sleep-interfering cognitions, such as overestimating and worrying about the negative consequences of insomnia and approaching bed time with fear of failure [30, 36].

#### Treatment of insomnia

The predominant treatment for insomnia is hypnotic drugs [37]. However, hypnotic drugs cause many side effects, including increased risk of falls resulting in fracture, cognitive impairment, impaired motor coordination, sedation, confusion, motor vehicle accidents, tolerance and dependence [38, 39], and are associated with increased mortality and risk of dementia [40–42]. In the search for non-pharmacological treatments for insomnia without severe side effects and with longer-lasting effects, clinical trials have tested the effects of exercise, thought control, imagery training, acupuncture, paradoxical intention, mindfulness and cognitive behavioural therapy for insomnia (CBT-i) [43–45]. Currently, non-pharmacological treatment for insomnia in patients with RA has focused only on physical activity and exercise training [46, 47].

However, hypnotic drugs do not address the precipitating events or perpetuating mechanisms that maintain chronic insomnia, and precipitating events or perpetuating mechanisms are not the primary focus or purpose of physical activity or exercise training. Furthermore, some patients can be disabled to a degree that inhibits them engaging in physical activity or exercise training. CBT-i is recommended as the first-line treatment for insomnia as it has demonstrated more effects than hypnotic drugs and has demonstrated a long-term effect [48–51]. In clinical settings, CBT-i is most often carried out by psychologists; however, treatment with CBT-i in groups led by nurses has been tested and the results showed significant positive effects [52, 53].

According to Morin and his model “The vicious circle of persistent insomnia”, the effect of CBT-i is caused by its ability to modify thought patterns and behaviours that reinforce poor sleep [54]. Thus, it is highly relevant to investigate the efficacy of CBT-i in patients with RA and, to our knowledge, this is the first clinical trial to investigate CBT-i in this population.

### Objectives {7}

Our primary objective is to compare the effect of CBT-i relative to usual care on changes in SE from baseline to week 7 in patients with RA.

Our key secondary objectives are to compare the effect of CBT-i relative to usual care on changes in sleep onset latency (SOL), wake after sleep onset (WASO), total sleep time (TST), insomnia, sleep quality, fatigue, impact of RA and depressive symptoms from baseline to week 26 in patients with RA.

Other secondary objectives are to compare the effect of CBT-i relative to usual care on changes in SE, SOL, WASO, TST, insomnia, sleep quality, fatigue, impact of RA, depressive symptoms, disease activity, tender joints, swollen joints, acute-phase reactant value, patient global assessment, physician global assessment, pain, functional status and HRQoL from baseline to weeks 7 and 26 in patients with RA.

### Trial design {8}

**Fig. 1:**
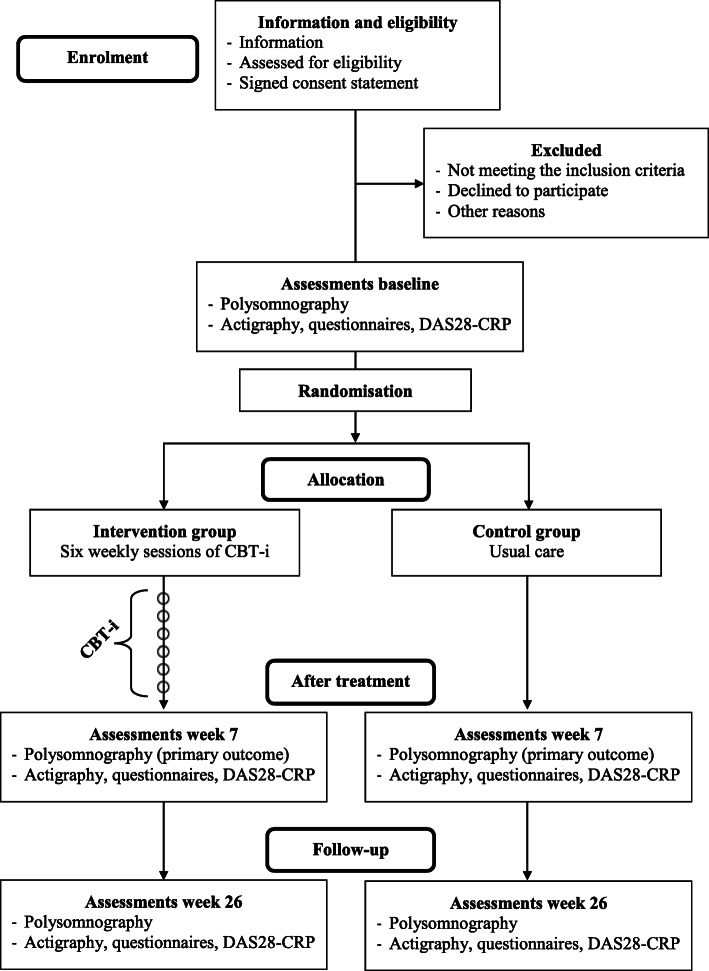
Study flowchart

The Sleep-RA trial will be carried out as a randomised controlled trial with a two-group parallel design. Participants (*n* = 60) will be allocated 1:1 to either CBT-i as treatment (intervention group) or to usual care (control group). Outcome assessments will be conducted three times for each participant: at baseline, after treatment (week 7) and at follow-up (week 26), as presented in Fig. 1.

## Methods: participants, interventions and outcomes

### Study setting {9}

We will recruit patients from hospital rheumatology outpatient clinics in The Capital Region of Denmark and Region Zealand are the official names of the two regions we recruite participants from.

### Eligibility criteria {10}

#### Inclusion criteria

Participants will be included if they: are diagnosed with RA according to the American College of Rheumatology/European League Against Rheumatism 2010 criteria [55]; have low to moderate disease activity, defined as DAS28-CRP ≤5.1 [4] and are 18 years of age or older; have both insomnia, defined as Insomnia Severity Index ≥11 [56] and chronic insomnia, according to the ICSD-3 duration criterion [29, 57]; have unchanged treatment with anti-rheumatic drugs for the preceding 3 months and no indication for changes of treatment with antirheumatic drugs during the intervention; and are able to understand and read Danish.

#### Exclusion criteria

Patients will be excluded if they: have had glucocorticoid administered by intramuscular, intra-articular or intravenous methods within the preceding 4 weeks; are on a continuous current daily intake of oral glucocorticoids >5 mg/day; have had a change in oral glucocorticoid dose within the preceding 3 months; have had prior or current professional non-pharmacological treatment for insomnia; work at night during the intervention, are pregnant or nursing a baby; have a current alcohol or drug-use disorder, according to their medical journal; have patient-related circumstances (physical or mental) that impede their ability to give informed consent and adhere to the programme.

### Who will take informed consent? {26a}

The corresponding author (KML) is the project manager of Sleep-RA and will obtain informed consent from participants.

### Additional consent provisions for collection and use of participant data and biological specimens {26b}

On the consent form, participants are asked to sign if they agree to have been given written and oral information on the purpose, method, advantages and disadvantages of saying yes to participate in the trial, and if they agree to participate voluntary. On the consent form, participants are informed that they can withdraw the consent without losing any rights to treatment, either current or future. They are also asked if they agree to the use of their data should they choose to withdraw from the trial.

This trial does not involve collecting biological specimens for storage.

### Interventions

Both groups will continually receive relevant treatment for their RA.

### Explanation for the choice of comparators {6b}

There are no standards on when and how to identify sleep disturbances in rheumatology outpatient consultations. Participants in the control group will receive usual care on sleep, which in the rheumatology outpatient clinics in The Capital Region of Denmark and in Region Zealand is sporadic and random and conducted via casual conversations based on the general knowledge of rheumatologists or nurses. Participants in the control group will be encouraged by KML to maintain their usual everyday lives and sleep patterns until follow-up assessment is conducted.

### Intervention description {11a}

In the Sleep-RA trial, CBT-i is a 6-week intervention with participant attendance of 2 h per week. The intervention will be conducted in groups of two to six participants per group. All sessions will be conducted by KML, who is a trained nurse in CBT-i.

The manual for the CBT-i intervention in Sleep-RA has been developed by KML, KBL and BAE in collaboration with a clinical psychologist trained in CBT-i and patient research partners, in accordance with the “Template for Intervention Description and Replication” checklist and guide [58]. The manual is guided by theory on CBT-i, available evidence and previous CBT-i manuals [54, 59–66]. The CBT-i intervention consists of sleep education, stimulus control, sleep restriction, cognitive therapy and relaxation, as presented in Table 1. The CBT-i intervention includes presentations by KML, exchanges of experiences, assignments, relaxation exercises and individual and shared discussions and reflections on how to use the knowledge gained during the intervention and changed habits related to sleep in daily life.

**Table 1:** Cognitive behavioural therapy for insomnia: intervention overview

**Session 1**	Introduction	Presentation
		Goals
		Expectations
	Sleep education	Normal sleep and its determinants
		Sleep needs
		Consequences of insomnia
		Nature of insomnia
		Evaluation of participant sleep
		Sleep hygiene
	Sleep restriction	Introduce sleep diary
**Session 2**	Follow-up	Since last session
	Sleep education	Sleep hygiene
	Stimuli control	Rationale; conditioned arousal
		Instructions
	Sleep restriction	Rationale; sleep drive and sleep consolidation
		Sleep efficiency and sleep window for week 2
	Relaxation	Bed time wind-down
**Session 3**	Follow-up	Since last session
	Stimuli control	Experiences and challenges
	Sleep restriction	Experiences and challenges
		Sleep efficiency and sleep window for week 3
	Cognitive therapy	Cognitions affecting sleep
		Cognitive techniques to treat insomnia
**Session 4**	Follow-up	Since last session
	Sleep restriction	Experiences and challenges
		Sleep efficiency and sleep window for week 4
	Cognitive therapy	Experiences and challenges
		Cognitive techniques to treat insomnia
		Worry-time
	Relaxation	Rationale, break the tension and return to a relaxed state
		Body scan meditation
**Session 5**	Follow-up	Since last session
	Stimuli control	Experiences and challenges
	Sleep restriction	Experiences and challenges
		Sleep efficiency and sleep window for week 5
	Cognitive therapy	Experiences and challenges
		Cognitive techniques to treat insomnia
	Relaxation	Experiences and challenges
**Session 6**	Follow-up	Since last session
	Sleep restriction	Experiences and challenges
		Sleep efficiency and sleep window for week 6
	Closure	Plan for further work
		Relapse prevention

### Criteria for discontinuing or modifying allocated interventions {11b}

If a participant in the intervention group is not able to participate in one CBT-i session due to unforeseen circumstances, an individual session is planned as soon as possible.

### Strategies to improve adherence to interventions {11c}

Adherence enhancement strategies are in use throughout the trial period, e.g. patients are informed of the importance of adhering regardless of allocation, and participants are offered reimbursements for travel [67].

#### Relevant concomitant care permitted or prohibited during the trial {11d}

Participants are prohibited from participating in any professional non-pharmacological treatment for insomnia other than Sleep-RA during the trial period.

### Provisions for post-trial care {30}

Participants allocated to the control group will be offered a nurse consultation in the rheumatology outpatient clinic with a focus on sleep when follow-up assessment is conducted.

### Outcomes {12}

#### Primary outcome

The primary outcome is SE at week 7 assessed by polysomnography (PSG) [68]. SE percentage = (TST/total time in bed (lights out to lights on)) × 100. In healthy persons, SE is 85–90%.

PSG is a comprehensive study of the biophysiological changes that occur in the human body during sleep. In Sleep-RA, 17 electrodes will be attached to each participant, recording data from electroencephalogram, eye movements electro-oculogram, electromyogram muscle activity or skeletal muscle activation, heart rhythm, respiratory airflow, respiratory effort and peripheral pulse oximetry [69]. All PSG studies will be conducted in the participants’ own homes. Data from PSG will be analysed in epochs of 30 s and managed at the Danish Center for Sleep Medicine (DCSM), Rigshospitalet, Glostrup.

#### Key secondary outcomes

**Fig. 2:**
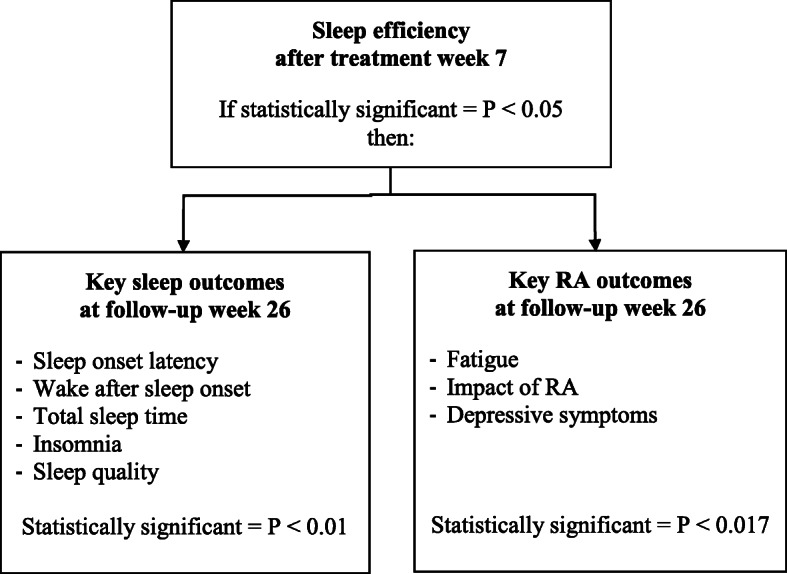
Statistical method for key secondary outcomes

We will examine the effect of improved sleep on key sleep outcomes and key RA outcomes at follow-up at week 26 if CBT-i has a significant positive effect on the primary outcome of SE after treatment at week 7, as presented in Fig. 2.

##### Key sleep outcomes

SOL will be assessed with PSG. SOL is the amount of time it takes to go from being fully awake to being asleep [70].

WASO will be assessed with PSG. WASO is the cumulative time awake between sleep onset and the final morning awakening [70].

TST will be assessed with PSG. TST is the cumulative time asleep between sleep onset and the final morning awakening [70].

Insomnia will be assessed with the Insomnia Severity Index questionnaire. This is a seven-item questionnaire designed to evaluate insomnia severity based on difficulties falling asleep, night-time awakenings, early morning awakenings, impairment of daytime functioning due to sleep problems, noticeability of impairments, distress or worry caused by sleep difficulties and dissatisfaction with sleep over the past 2 weeks. Each item is rated using a five-point Likert scale ranging from 0 (not at all) to 4 (very much), for a total score ranging from 0 (no insomnia) to 28 (clinical insomnia) [71]. A cut-off of 11 has been validated in a clinical sample of people with insomnia and demonstrated a sensitivity of 97.2% and a specificity of 100% [56].

Sleep quality will be assessed with the Pittsburgh Sleep Quality Index (PSQI) questionnaire. The PSQI measures self-reported sleep over the past 4 weeks [72]. It includes nine items/18 questions with seven categories, including subjective sleep quality, sleep latency, sleep duration, habitual SE, sleep disturbances, use of sleep medications and daytime dysfunction. A global sum score for the PSQI >5 indicates poor sleep. The Cronbach’s α value for the PSQI is 0.83 [73].

##### Key RA outcomes

Fatigue will be assessed with the Bristol Rheumatoid Arthritis Fatigue Multidimensional Questionnaire (BRAF-MDQ) for the previous 7 days [74]. The BRAF-MDQ consists of 20 items with the opportunity to define five domains: total fatigue, physical fatigue, living with fatigue, cognitive fatigue and emotional fatigue. The total score ranges from 0 to 70, with a higher score indicating more severity. The Cronbach’s α value for BRAF-MDQ is >0.93 [75].

The impact of RA on daily life will be assessed with the Rheumatoid Arthritis Impact of Disease (RAID) questionnaire [76, 77]. RAID consists of seven items, each of which is rated using a numerical rating scale of 0–10 with higher scores indicating more severity, covering pain, physical function, fatigue, sleep, physical well-being, mental well-being and coping for the previous 7 days. The Cronbach’s α value for RAID is >0.93 [75].

Depressive symptoms will be assessed with the Hospital Anxiety and Depression Scale-Depression (HADS-D) [78]. HADS-D measures depressive symptoms in the previous 7 days. Seven items are rated on a four-point scale and scored from 0 to 3, with total scores ranging from 0 to 21. Scores between 0 and 7 represent ‘no case’, 8 to 10 indicate ‘possible case’, 11–15 ‘moderate case’ and ‘15–21’ suggest a ‘probable severe case of depression’ [79]. The Cronbach’s α value for HADS-D is 0.82 [80].

#### Other secondary outcomes

We will examine other secondary outcomes after treatment at week 7 and at follow-up at week 26 if CBT-i has a significant positive effect on the primary outcome of SE after treatment at week 7.

##### Other sleep outcomes

SE, SOL, WASO and TST will also be assessed with actigraphy to measure sleep objectively for 7 continuous nights. Actigraph is an objective measurement to assess sleep/wake behaviour for several continuous nights and days [81, 82]. It allows the participant to be mobile and to continue their normal routines while data are recorded in a natural sleep environment. In Sleep-RA, the actigraph is worn on the non-dominant wrist for 7 continuous nights and days. To validate or adjust the data from the actigraph, the participants write down their bed times and rise times and the use of the actigraph.

SE, SOL WASO and TST will be further assessed subjectively with the PSQI [72, 73].

##### Other RA outcomes

Fatigue severity, coping and effect over the previous 7 days will be assessed with the Bristol Rheumatoid Arthritis Fatigue Numerical Rating Scale (BRAF-NRS) on a scale of 0–10, with higher scores indicating greater severity. BRAF-NRS has shown good criterion and construct validity [83].

RA-related pain will be assessed with a self-reported visual analogue scale (VAS) for pain on a scale of 0–100, with a higher score indicating greater severity [84].

Functional status will be assessed with the Multidimensional Health Assessment Questionnaire (MD-HAQ) [85]. The MD-HAQ measures self-reported functional status over the previous 7 days on 10 items covering dressing, rising, eating, walking, hygiene, reach, grip and everyday activities. The mean of the MD-HAQ score is calculated with a possible range of 0.0–3.0, with a higher score indicating lower functional status. The MD-HAQ is validated for patients with RA and has a Cronbach’s α value of 0.65 for the psychological dimension and 0.88 for the physical dimension [86].

HRQoL will be assessed with the Short Form-36 health survey (SF-36) [87, 88]. This is a generic instrument consisting of 36 multiple-choice questions contributing to the sum scores of physical and mental health. The two scores of 0–100 are the weighted sums of the items in their section, with a higher score indicating less disability. The SF-36 is validated and translated to Danish. The Cronbach’s α value for SF-36 is >0.85 [89].

Disease activity will be assessed with DAS28-CRP. DAS28-CRP is a composite index developed to make an objective and reproducible assessment of RA disease activity and has been validated for use in clinical trials [90, 91]. A DAS28-CRP <2.6 implies remission, ≥2.6 and ≤3.2 implies low disease activity, >3.2 and ≤5.1 implies moderate disease activity, and >5.1 implies high disease activity.

Tender and swollen joints will be assessed by a physical examination of 28 joints, conducted by project nurses [92].

CRP will be assessed from serum samples. A higher CRP value indicates more inflammation [91, 93].

The patient global assessment of disease activity is a self-reported assessment of the patient’s overall assessment of how the arthritis is doing with the question: “Considering all the ways your arthritis affects you, rate how well you are doing on the following scale?” [94]. The patient reports “VAS global patient” from 0 to 100, with a higher score indicating more disease activity [95].

The physician global assessment is a well-validated assessment of the patient’s current disease activity [94]. The project nurses will report “VAS global physician” from 0 to 100, with a higher score indicating more disease activity [95].

#### Additional information

Additional information includes sociodemographic characteristics (education, employment, civil status, children living at home), lifestyle (smoking, alcohol, drugs, caffeine, physical activity), RA-related information (duration of RA, rheumatoid factor of the immunoglobulin M class, anti-citrullinated protein antibody, CRP), sleep-related information (duration of insomnia, sleep stages, number of awakenings, apnoea-hypopnoea index, leg movements), comorbidity (diabetes, hypertension, heart attack, other heart disease, stroke, chronic obstructive pulmonary disease, cancer, osteoarthritis, osteoporosis, asthma, depression) and current medicine (antirheumatic drugs, glucocorticoids, antidepressant drugs, hypnotic drugs).

### Participant timeline {13}

The trial period for each participant is approximately 6 months, and includes 6 weeks of CBT-i for participants in the intervention group and 20 weeks of subsequent outcome assessments for both groups. All participants will complete the same outcome assessments at baseline, after treatment (week 7) and at follow-up (week 26), as presented in Fig. 1 and Table 2.

**Table 2:**
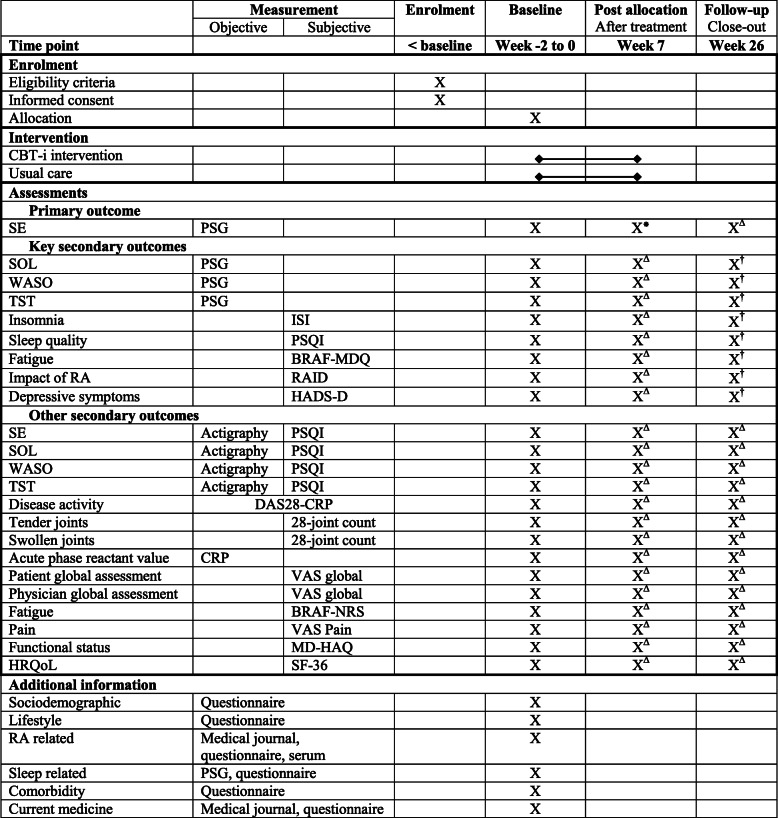
Standard Protocol Items: Recommendations for Interventional Trials (SPIRIT) trial schedule

*Primary outcome; ^†^key secondary outcomes; ^Δ^other secondary outcomes

*BRAF-MDQ* Bristol Rheumatoid Arthritis Fatigue Multidimensional Questionnaire, *BRAF-NRS* Bristol Rheumatoid Arthritis Fatigue Numerical Rating Scale, *CBT-i* cognitive behavioural therapy for insomnia, *CRP* C-reactive protein, *DAS28* Disease Activity Score-28, *HADS-D* Hospital Anxiety and Depression Scale-Depression, *HRQoL* health-related quality of life, *ISI* Insomnia Severity Index, *MDHAQ* Multidimensional Health Assessment Questionnaire, *PSG* polysomnography, *PSQI* Pittsburgh Sleep Quality Index, *RA* rheumatoid arthritis, *RAID* Rheumatoid Arthritis Impact of Disease, *SE* sleep efficiency, *SF-36* Short Form-36 health survey, *SOL* sleep-onset latency, *TST* total sleep time, *VAS* visual analogue scale, *WASO* wake after sleep onset

### Sample size {14}

With a total sample size of 60 patients with RA (30 allocated to CBT-i as treatment and 30 allocated to usual care), we will have more than 85% power to detect a group difference in the primary outcome of average SE assessed by PSG after treatment at week 7 and subsequently with reasonable power in the key secondary outcome assessed at follow-up at week 26.

For a two-sample pooled *t* test of a normal mean difference with a two-sided significance level of 0.05 (*P* < 0.05), assuming a common standard deviation of 7.5% in SE, a total sample size of 60 with allocation ratio of 1:1 has a power of 86.1% to detect a mean difference of 6 percentage points in SE.

The Sleep-RA trial is sufficiently powered to detect the anticipated difference with a total sample size of 52 participants. Assuming a balanced attrition rate (no more than four withdrawals per group) we will obtain enough statistical power (80.7%, i.e. >80%) to detect a mean difference of 6 percentage points in SE.

With a total sample size of 60 patients and assuming a balanced design (1:1), exploratory analyses of any of the secondary outcomes collected at week 26 will have enough statistical power (>80%) to detect a standardised mean difference of 0.80 (i.e. a Cohen’s effect size corresponding to a large clinical effect).

### Recruitment {15}

All patients at routine rheumatologic outpatient visits in Denmark enter self-reported data on a touch screen linked to the National Clinical Database for Rheumatology (DANBIO) [96]. Potential participants for Sleep-RA will be identified when they answer the question: “At this moment, are you able to get a good night’s sleep?” Patients who answer either “With much difficulty” or “Unable to” will be presented with a pop-up message on the screen containing brief information that a research project on sleep is underway (and currently recruiting participants). Patients who want further information can type “yes” on the screen. KML will telephone each patient and will offer to send written information and subsequent oral information. While giving the oral information, it will be determined whether the patient fulfils the criteria and can enter the trial.

Patients who are not eligible or do not wish to participate in the trial will be registered in one of the following three categories: 1) did not meet the inclusion criteria; 2) declined to participate; or 3) other reasons (Fig. 1).

### Assignment of interventions: allocation

#### Sequence generation {16a}

Randomisation will be conducted after baseline assessment by computer-generated random numbers with blocks of ten participants.

During randomisation, participants will be stratified by disease activity to equalize the risk of change in treatment with antirheumatic drugs during the trial period and the effect this might have on sleep. Stratification will be set to disease activity according to DAS28-CRP ≤3.2 (low disease activity) and >3.2 (moderate disease activity) measured at baseline [97].

#### Concealment mechanism {16b}

Allocation concealment will be ensured as it will not be possible to conduct the computer-generated randomisation until the baseline assessment is completed for each participant.

#### Implementation {16c}

An external collaborator (Zitelab Aps, http://www.zitelab.dk/) will generate the allocation sequence. This collaborator is not involved in outcome assessments or the CBT-i intervention. KML will enrol participants and, immediately after randomisation, inform them to which group they have been allocated.

### Assignment of interventions: blinding

#### Who will be blinded {17a}

Outcome assessments are conducted by project nurses specialising in rheumatology, medical laboratory technologists or neurophysiology assistants, all of whom will be blinded to participant allocation.

Application of PSG equipment and instructions on how to use the actigraph and subsequent analysis of data will be conducted by neurophysiology assistants at the DCSM. Patient-reported outcome measures will be collected via questionnaires on tablets handed out by a project nurse. To assess the outcome of disease activity, a project nurse will examine the joints of participants to identify the number of swollen and tender joints related to DAS28. Blood samples will be taken and analysed by a medical laboratory technologist.

KML will conduct the CBT-i intervention and is therefore not blinded to participant allocation.

#### Procedure for unblinding if needed {17b}

The trial design is open-label with only the outcome assessors being blinded so unblinding will not occur.

### Data collection and management

#### Plans for assessment and collection of outcomes {18a}

Data will be collected from PSG, actigraphy, physical examination, blood samples, a medical journal and questionnaires as presented in Table 2 and as follows.

The data collected from PSG cover SE, SOL, WASO, TST, sleep stages, number of awakenings, apnoea-hypopnoea index and leg movements. The data collected from actigraphy cover SE, SOL, WASO and TST for 7 continuous nights. From the medical journal we will collect data on duration of RA, anti-rheumatic drugs and glucocorticoids. Questionnaires will provide data on patient-reported SE, SOL, WASO, TST, insomnia, sleep quality, fatigue, impact of RA, depressive symptoms, pain, functional status, HRQoL, patient global assessment, physician global assessment, sociodemographic information, lifestyle, RA-related information, comorbidity, duration of insomnia, antidepressant drugs and hypnotic drugs. From the physical examination we will obtain data on swollen joints and tender joints counts (of 28 examined joints), and we will collect data on CRP from serum samples.

#### Plans to promote participant retention and complete follow-up {18b}

Dates for assessments will be planned by KML together with each participant, and all participants will receive a text reminder on their telephone or an email the day before a scheduled assessment. If participants withdraw consent for follow-up assessment of one outcome, they will be given the option to continue with assessments for other outcomes.

#### Data management {19}

All participants will receive an identification code and all data will be de-identified.

A list of identifiable participant information associated with each identification code will be stored electronically separately from the research data for 5 years. Patient-reported information will be completed electronically on a tablet, which will be used only for participants in this trial and is linked to the DANBIO Reuma electronic case report form.

#### Confidentiality {27}

All information collected during the trial will be kept confidential in accordance with Danish Data Protection Agency rules. All data will be processed confidentially according to the Act on Processing of Personal Data.

#### Plans for collection, laboratory evaluation and storage of biological specimens for genetic or molecular analysis in this trial/future use {33}

There will be no biological specimens collected for genetic or molecular analysis in this trial.

### Statistical methods

#### Statistical methods for primary and secondary outcomes {20a}

All main analyses will be conducted based on the intention-to-treat (ITT) population. We plan to conduct both an analysis of the full analysis set (patients with outcome data available at baseline) and a per-protocol analysis so that any differences between them can be explicitly discussed and interpreted. The ITT principle states the effect of a treatment policy (i.e. the planned treatment regimen) rather than the actual treatment given. The consequence of conducting ITT analyses is that participants allocated to a treatment group should be followed up, assessed and analysed as members of that group, irrespective of their compliance to the planned course of treatment. Robustness is a concept that refers to the sensitivity of the overall conclusions to various limitations of the data, assumptions and analytic approaches to data analysis. Robustness implies that the treatment effect and primary conclusions of the trial are not substantially affected when analyses are carried out based on alternative assumptions or analytical approaches.

We will control the α level across only the most important hypotheses by use of a hierarchical testing strategy to preserve a type 1 error rate of 5% for the primary outcome of change in SE assessed after treatment at week 7, followed by SE assessed at follow-up after 26 weeks. All the patient-reported outcome measures and other secondary outcome measures will, by principle, only be clinically relevant after 26 weeks. The sleep-related key secondary outcomes (SOL, WASO, TST, insomnia and sleep quality) will only be considered statistically significant with a *P* value <0.01 (0.05/5). The RA-related key secondary outcomes (fatigue, impact of RA and depressive symptoms) will only be considered statistically significant with a *P* value <0.017 (0.05/3) as described in Fig. 2.

In the Sleep-RA trial with repeated measures, participants will be randomly assigned to treatment groups, and outcome observations are made at two time points on each patient. We anticipate that measures on the same patient at different times are correlated and that measures taken close together in time will be more highly correlated than measures taken further apart in time; observations on different patients will be assumed to be independent.

Data will be analysed using the PROC MIXED procedure of the statistical program SAS System, with baseline level as a covariable, using a multilevel repeated-measures random-effects model, with participants as the random effect factor and based on a restricted maximum likelihood estimate. For the primary outcome measure, the after-treatment value will be the response variable, and the baseline values of treatment group (two levels), stratum (i.e. two levels according to the randomisation) and time (two levels) will be the covariates. Assessment of these baseline values (main effects) will be of interest, along with the interaction between treatment group and time. This statistical model holds all between-group comparisons at both assessment points and allows for evaluation of the average effect over the period from baseline to follow-up at 26 weeks.

The SAS statistical package (v.9.4; SAS institute Inc., Cary, NC, USA) and R 3.0.1 (http://www.R-project.org, the R Foundation for Statistical Computing) will be used for the statistical models.

#### Interim analyses {21b}

We plan to include 60 patients, and the trial period for each participant is 26 weeks. This is a non-pharmacological 6-week intervention with no expected adverse or harmful events, and the trial is therefore not subject to independent safety monitoring and periodical review, e.g. interim analyses.

#### Methods for additional analyses (e.g. subgroup analyses) {20b}

We have no intention to conduct subgroup and adjusted analyses.

#### Methods in analysis to handle protocol non-adherence and any statistical methods to handle missing data {20c}

We will apply the analysis framework suggested by White et al. for ITT analysis that depends on making plausible assumptions about the missing data and including all participants in sensitivity analyses [98]. We will:

1. Attempt to follow-up all randomised participants, even if they withdraw from allocated treatment.
2. Perform a main analysis of all observed data that are valid under a plausible assumption about the missing data (missing at random). Our primary analysis population will be all participants with available data statistically modelled using repeated-measures linear mixed models (see below). These models will be valid if data are missing at random (any systematic difference between the missing values and the observed values can be explained by differences in observed data).
3. Perform sensitivity analyses to explore the effect of departures from the assumption made in the main analysis (data as observed; valid assuming that data are missing completely at random).
4. Account for all randomised participants, at least in the sensitivity analyses. We will analyse all variables with missing data (due to withdrawals) being replaced by imputation of the baseline level, i.e. interpreted as assuming that those who dropped out returned to their baseline level [99]. These models will potentially be valid even if data are ‘missing not at random’ (even after the observed data are considered, systematic differences remain between the missing values and the observed values).

In addition, data will be analysed according to the per-protocol population, where data from participants who participated in a minimum of four out of six sessions of CBT-i are included. The participants from the control group will be considered per-protocol individuals if data are available from all three assessments (baseline, after treatment at week 7, and follow-up at week 26).

#### Plans to give access to the full protocol, participant-level data and statistical code {31c}

The datasets analysed during this trial are available from the corresponding author on reasonable request.

### Oversight and monitoring

#### Composition of the coordinating centre and trial steering committee {5d}

This trial proceeds from COPECARE (Copenhagen Center for Arthritis Research), a multidisciplinary centre which is internationally renowned and provides day-to-day support for the trial. COPECARE represents the steering group in collaboration with the University Hospitals Centre for Nursing and Care Research, Parker Institute and DCSM.

The steering committee consists of health professionals with solid research and management competences. The committee represents knowledge at a high level from different professions: nurses, rheumatologists, psychologist, neurophysiologist and statistician. This ensures scientific quality, multidisciplinary width and possible implementation in clinical practice depending on the results of the trial. The managers of the rheumatology department participate in the steering committee and ensure clinical anchoring and implementation.

Once a year an external assessor from the University of Copenhagen is invited to a meeting with the project management group to discuss progress in the trial and methods to improve quality and stringency.

The daily project manager (KML) is affiliated to COPECARE. She identifies all potential participants from the recruiting sites through DANBIO (as explained in the “Recruitment” section). She provides oral information and received written consent.

#### Composition of the data monitoring committee, its role and reporting structure {21a}

The intervention in this trial is a non-pharmacological intervention with low or no risk of adverse or harmful events. Therefore, there is no data monitoring committee. The trial is registered on www.clinicaltrials.gov and approved by the Regional Ethics Committee and the Danish Data Protection Agency.

#### Adverse event reporting and harms {22}

Participants will be monitored throughout the period of the intervention to detect any unintended events.

Should a PSG show signs of a sleep disorder requiring further examination or treatment the participant will be contacted by DCSM after the final assessment is completed.

#### Frequency and plans for auditing trial conduct {23}

The project management group meet twice a year and conduct an audit of the trial.

When a participant has completed self-reported questionnaires on the tablet used in this trial, the project nurse ensures that data are complete and imported to the electronic case report form. When objective sleep measures of PSG and actigraphy are completed, all data are assessed for completeness by a neurophysiology assistant. If any data are missing or of low quality, the participant is invited to a new assessment.

#### Plans for communicating important protocol amendments to relevant parties (e.g. trial participants, ethical committees) {25}

Important protocol amendments which may impact the content or conduct of the trial will be decided in the steering group. Important protocol amendments will be sent for approval to the ethics committee, notified to the participating sites and addressed in the written and oral information. Furthermore, the protocol registered on www.clinicaltrials.gov will be updated prior to implementation.

### Dissemination plans {31a}

We plan to publish two scientific papers in peer-reviewed journals based on the trial and to disseminate the results to patient organisations and the public through printed and electronic media.

## Discussion

The Sleep-RA trial is designed to investigate the efficacy of CBT-i on sleep and prevalent symptoms of fatigue, impact of RA, depressive symptoms, pain, functional status and HRQoL in patients with RA. To our knowledge, this is the first trial to investigate an evidence-based non-pharmacological intervention that, first and foremost, targets sleep-related cognitions and behavioural challenges related to sleep disturbances in this population.

Patients with RA have increased risk of fatigue, depressive symptoms, pain, reduced functional status and work capability. The severity of these symptoms is often linked to the disease activity, and patients with symptoms report that they feel constantly reminded of their chronic disease. The degree and duration of the above-mentioned symptoms is unpredictable because of the fluctuating character of RA. Living with one or more of these symptoms or the awareness that they can occur unexpectedly at any time can challenge the patient’s ability to manage the arthritis and their daily life. According to Spielman’s conceptual “3P model” of insomnia, insomnia is caused by precipitating events such as a change in health [100, 101]. Therefore, it is possible that there is an increased risk of initial insomnia around the time of diagnosis or when patients have arthritis flares and experience symptoms, while not knowing how long they will last. The development of chronic insomnia is complex, and is mainly—if not solely—mediated by perpetuating mechanisms [30]. RA symptoms are unpredictable, and because sleep disturbances are also associated with many of the same symptoms as RA, e.g. fatigue, depressive symptoms, pain and HRQoL, this could trigger, maintain and extend perpetuating mechanisms. In the persistent search for better sleep quality and more sleep, patients can develop a hyper-focus on their lack of sleep. This often increases the patient’s risk of obsession with sleep, followed by cognitions and behaviour that work against falling asleep [30, 36].

A positive effect of CBT-i will document CBT-i to be an applicable and pertinent treatment of insomnia in patients with RA, as recommended in international guidelines for non-pharmacological treatment of insomnia [62]. In addition, if the trial also finds positive effects regarding the secondary outcomes, such as fatigue, depressive symptoms, pain, disease activity and functional status, CBT-i will not only be relevant as a non-pharmacological treatment option for insomnia but will also have the potential to contribute positively in the management of symptoms related to RA and insomnia.

The primary outcome in Sleep-RA is SE which can be measured in different ways, e.g. with PSG, actigraphy, PSQI and a sleep diary. Although PSG is perceived as the most valid measure for SE [69], we are aware that PSG could inconvenience the participants in terms of the application of electrodes. Nevertheless, we found it necessary to ensure that a measuring method of high quality was used to generate results and because Sleep-RA is the first trial to examine CBT-i in patients with RA.

CBT-i is most often carried out by psychologists or other professionals with training in psychology in private clinics. Consequently, CBT-i is generally not accessible at hospitals, either as a part of the patient’s current treatment for RA or in the management of their symptoms. It is highly relevant to investigate the effect of nurse-led CBT-i in patients with RA since significant positive effects of CBT-i delivered in a group format by nurses have been shown in randomised controlled trials in European general practice settings [52, 53].

For future clinical purposes, we expect positive results from Sleep-RA to contribute to increased awareness of insomnia in rheumatology clinical practice. CBT-i has the potential to contribute significantly to the treatment of insomnia and, consequently, CBT-i has the potential to reduce the prevalence of chronic insomnia. Many symptoms of RA and insomnia are identical and some of them can be challenging to treat. CBT-i may also have comprehensive positive effects on the wide range of symptoms and thereby potentially contribute to increased physical and mental health and well-being in patients with RA.

## Trial status

This is protocol version 1, 16 January 2020.

Initial release was on 30 November 2018. Recruitment started on 17 December 2018, with an anticipated primary completion date of 30 October 2020.

## Data Availability

Any data required to support the protocol can be supplied on request.

## Abbreviations

BRAF-MDQ: Bristol Rheumatoid Arthritis Fatigue Multidimensional Questionnaire
BRAF-NRS: Bristol Rheumatoid Arthritis Fatigue Numerical Rating Scale
CBT-i: Cognitive behavioural therapy for insomnia
CRP: C-reactive protein
DANBIO: The National Clinical Database for Rheumatology
DAS28: Disease Activity Score-28
DCSM: Danish Center for Sleep Medicine
HADS-D: Hospital Anxiety and Depression Scale-Depression
HRQoL: Health-related quality of life
ICSD-3: International Classification of Sleep Disorders third edition
ITT: Intention-to-treat
MD-HAQ: Multidimensional Health Assessment Questionnaire
PSG: Polysomnography
PSQI: Pittsburgh Sleep Quality Index
RA: Rheumatoid arthritis
RAID: Rheumatoid Arthritis Impact of Disease
SE: Sleep efficiency
SF-36: Short Form-36 health survey
SOL: Sleep onset latency
TST: Total sleep time
VAS: Visual analogue scale
WASO: Wake after sleep onset
